# Feasibility of a Web-Based Platform (Trial My App) to Efficiently Conduct Randomized Controlled Trials of mHealth Apps For Patients With Cardiovascular Risk Factors: Protocol For Evaluating an mHealth App for Hypertension

**DOI:** 10.2196/26155

**Published:** 2021-02-01

**Authors:** Cynthia Lokker, Rita Jezrawi, Itzhak Gabizon, Jobin Varughese, Michael Brown, Dan Trottier, Elizabeth Alvarez, Jon-David Schwalm, Michael McGillion, Jinhui Ma, Vinai Bhagirath

**Affiliations:** 1 Health Information Research Unit Department of Health Research Methods, Evidence, and Impact McMaster University Hamilton, ON Canada; 2 Department of Cardiology Soroka University Medical Center Ben-Gurion University of the Negev Beer Sheva Israel; 3 Department of Family Medicine Faculty of Health Sciences McMaster University Hamilton, ON Canada; 4 Queen Square Family Health Team Brampton, ON Canada; 5 Department of Health Research Methods, Evidence, and Impact McMaster University Hamilton, ON Canada; 6 Population Health Research Institute Hamilton Health Sciences McMaster University Hamilton, ON Canada; 7 Centre for Health Economics and Policy Analysis McMaster University Hamilton, ON Canada; 8 School of Nursing Faculty of Health Sciences McMaster University Hamilton, ON Canada; 9 Department of Medicine Faculty of Health Sciences McMaster University Hamilton, ON Canada

**Keywords:** mHealth, mobile health, hypertension, app, patient-oriented, feasibility, cardiovascular disease, internet-administered, randomized controlled trial

## Abstract

**Background:**

Mobile health (mHealth) interventions can improve health by improving cardiovascular risk factors, but their adoption in care by physicians and patients is untapped. Few mHealth apps have been evaluated in clinical trials, and due to the fast pace of technological development, those previously evaluated are often outdated by the time trial results are available. Given the rapid pace of change in this field, it is not feasible to rigorously evaluate mHealth apps with current methodologies.

**Objective:**

The overall aim of this pilot study was to test the feasibility of using a web research platform called Trial My App to conduct efficient and rigorous web-based randomized controlled trials (RCTs) of mHealth apps relevant to patients with cardiovascular risk factors by evaluating an app that targets hypertension.

**Methods:**

For this study, 200 participants with suboptimally controlled hypertension will be recruited through advertisements in newsletters, media, and the internet, as well as through referrals from their health care providers. Screening, consent, randomization, and collection of patient-important health confidence and self-management ability outcomes will be conducted online through the Trial My App research platform. Participants will be randomized into 2 groups: 100 that will use an mHealth app for tracking hypertension and 100 that will be considered as an educational control. All participants will complete questionnaires at 0, 1, 3 and 6 months after enrolment. A substudy to validate the method of blood pressure readings and the consistency of data entered through Trial My App will be conducted with 40 participants.

**Results:**

The development of the Trial My App web platform has been completed. The creation of survey instruments has been completed in collaboration with our patient partners and advisory board. Recruitment is expected to begin in the first quarter of 2021; data collection and analysis are expected to be completed approximately 1 year after study commencement. Results will be disseminated through conferences and publications. The primary outcomes of this study include the feasibility of conducting an RCT using the Trial My App platform by reporting recruitment, retention, and completion statistics. We will validate app-entered data with a standard 7-day home blood pressure measurement method. Lastly, the pilot, nonblinded RCT will assess the effectiveness of the mHealth app in improving the control of hypertension compared with the control of hypertension in the educational control group.

**Conclusions:**

This study will determine if it is feasible to use the Trial My App web-based platform to evaluate the effectiveness of mHealth apps for patients with cardiovascular risk factors. As more mHealth apps are evaluated in RCTs, patients will be able to select apps that meet their needs and physicians will be able to make evidence-based recommendations to their patients for apps aimed at improving cardiovascular health.

**Trial Registration:**

ClinicalTrials.gov NCT04528654; https://clinicaltrials.gov/ct2/show/NCT04528654

**International Registered Report Identifier (IRRID):**

PRR1-10.2196/26155

## Introduction

Smartphones provide continuous connection to the internet and can run sophisticated software apps. Globally, over 3.5 billion individuals own a smartphone [1]. The delivery of health care interventions via mobile phones is known as mobile health (mHealth). In 2017, 86% of the Canadians surveyed owned a smartphone or tablet and 78% downloaded mHealth and other types of apps to these devices [2]. Of the 66% of Canadians who indicated that they self-track at least one aspect of their health, 40% used electronic devices to do so and 32% reported using at least one mHealth app to monitor their health [2,3]. Other authors have reported that up to 58% of the smartphone users have downloaded an mHealth app [4] and 3.6 billion health apps were projected to be downloaded in 2017 [5]. Since smartphones are used to track health and are often continuously carried by users, mHealth apps allow for frequent data collection and feedback for behaviors that affect health, and interventions can be deployed to many users at a relatively low cost [6].

Health behaviors, including smoking, inactivity, and poor diet, are the major contributors to cardiovascular disease [7]. The American Heart Association has endorsed provider and patient self-management of cardiovascular disease risk factors as an effective form of secondary prevention [8]. Though the field of mHealth is in its infancy, early studies have shown that apps can improve health-related behaviors and reduce cardiovascular risk factors, largely through knowledge translation, by improving adherence to or by uptake of medications and behaviors that are known to be effective [9]. Randomized controlled trials (RCTs) report that apps can help patients lose weight [10-14], quit smoking [15], and increase physical activity [16]. An RCT of lifestyle-focused text messages reduced low-density lipoprotein cholesterol levels, systolic blood pressure, and BMI in patients with coronary heart disease [17], and a systematic review of RCTs of home blood pressure telemonitoring showed reduced systolic and diastolic blood pressure [18,19]. Thus, current data suggest that mHealth interventions can improve health by improving such cardiovascular risk factors, but their adoption in health care by physicians and patients is minimal. The 2014 National Physician Survey of licensed Canadian physicians reported that 83% did not recommend mobile apps to their patients, although 72% of the general practitioners and 53% of the specialists referred patients to websites and 50% used mobile apps such as e-textbooks or calculators in their practices [20]. We recently conducted a needs assessment survey of 113 physicians, which showed that over half of the physicians recommended apps to their patients despite their lack of clinical evidence. Many physicians in the survey indicated that they rely on personal opinions and patient recommendations to support their recommendations of apps despite wanting to choose apps that have a higher level of evidence such as RCTs or expert panel reviews. Our needs assessment also indicated that physicians do not recommend apps because they are not aware of them or do not have time to review their clinical efficacy. Without proper testing and evaluation, gaps in app development, including lack of expert involvement, poor user input validation, lack of evidence base, and poor quality of information, may pose clinical risks and safety harms to consumers [21]. With the growing interest in mHealth to monitor patient health, it is imperative that physicians stay informed and minimize their medical-legal risks by recommending apps with proven efficacy.

Despite this desire for better evidence, only a minority of the mHealth apps available in web-based app stores and commonly downloaded by patients have been evaluated in clinical trials, and due to the fast pace of technological development, those that have been evaluated are often outdated by the time trial results are available. Of the top 100 grossing health and fitness apps, researchers found that none had been formally evaluated in clinical studies [22]. Multiple reviews have highlighted the lack of quality research evidence on the efficacy of apps [9,23-25]. mHealth research has several unique challenges contributing to this problem. Chiefly, technology is rapidly progressing and equally rapid techniques for evaluating such technology are needed. Testing must be cost-efficient, given the limited funding for evaluating mHealth interventions compared with pharmacological interventions or medical devices [9]. These constraints limit the evidence base and the incorporation of apps in patient care.

The rapid proliferation of smartphone technology provides untapped potential to improve the efficient conduct of such research. Using internet-enabled devices to perform research can potentially (1) accelerate large-scale enrolment by contacting and screening potential participants who do not frequently interact with the health system through clinics or hospitals and (2) reduce costs and improve participation by allowing frequent and inexpensive data collection directly from participants. With the rapid development of internet-connected and smartphone-connected consumer devices that can collect biometric data, smartphones also have the potential to collect objective, real-time data directly from patients [26].

## Methods

### Overview

We have developed an innovative research approach using a web-based platform called Trial My App, which is designed to perform efficient trials of apps relevant to patients with cardiovascular risk factors. In our initial phase, we engaged an advisory board of patients to codevelop criteria for app and trial outcome selection, which will be used to support further deployment of Trial My App. The patients used apps for goal setting, decision-making, information sharing, and empowerment in managing their health. From these themes, we derived a series of survey questions to determine if an app was meeting these outcomes, and we included this survey in the pilot trial. Content analysis by the advisory board also indicated that patients considered a number of favorable technical factors when selecting their mobile apps: (1) relevant feedback on progress, (2) security, (3) low cost, (4) customizability, (5) usability, (6) information credibility, (7) multifunctional app integration, and (8) interdevice compatibility. The undesired features were as follows: (1) unreliable technology, (2) distraction, (3) collection of personal information, and (4) learning curve. These criteria were applied to shortlist apps for hypertension, and through discussion with the research team and patient partners, we identified Sphygmo BP as the intervention app for this pilot trial (Multimedia Appendix 1). Sphygmo BP was created in partnership with the University of Alberta to help patients with hypertension to self-manage their blood pressure. The app tracks and averages blood pressure, glucose levels, weight, temperature, respiratory rate, and oxygen saturation. It also includes educational components to facilitate better patient awareness of management strategies for blood pressure and is designed to facilitate patient-physician communication through telemonitoring.

### Objectives

The primary objective of the study is to test the feasibility of conducting an RCT of an mHealth hypertension-tracking app using the Trial My App platform. The secondary objective is to test if the use of the Sphygmo BP app reduces blood pressure in patients with suboptimally controlled hypertension when compared with the use of a website with information on hypertension. It would be valuable for patients and physicians to know whether the use of the app is likely to result in reductions in blood pressure that have been shown to reduce clinical outcomes.

### Study Design

This is a pilot, nonblinded parallel-group RCT, comparing the use of a hypertension app versus an education control in participants with suboptimally controlled hypertension. Outcomes include feasibility, clinical, and patient-important endpoints.

### Eligibility Criteria

The inclusion criteria are as follows: (1) age over 18 years, (2) diagnosis of hypertension, (3) interested in using an app for hypertension management, (4) access to a smartphone with internet connection, and (5) access to a blood pressure monitoring device (in home or community setting, eg, pharmacy). The exclusion criteria are as follows: (1) participant-reported blood pressure within target (target range for patients with diabetes is systolic blood pressure <130 mm Hg and diastolic blood pressure <80 mm Hg; for those without diabetes, target range is systolic blood pressure <140 mm Hg and diastolic blood pressure <90 mm Hg according to Hypertension Canada guidelines [27]) within the 2 weeks prior to enrolment, (2) emergent hypertensive concerns (potential participants with systolic blood pressure ≥180 mm Hg or diastolic blood pressure ≥120 mm Hg will be advised to seek medical attention and will be excluded), (3) current use of a mobile app for hypertension management, (4) living outside of Canada, (5) pregnancy, and (6) unwillingness or inability to give informed consent.

### Recruitment

The primary study site will be the Health Information Research Unit at McMaster University in Hamilton, Ontario. A combination of passive and active recruitment strategies will be used. A variety of recruitment materials will be distributed by a research assistant throughout the community and outpatient or specialty clinic waiting rooms. These materials include videos, papers, and web-based posters/postcards, emails, as well as posts advertising on social media. Social media recruitment on Facebook, Twitter, and Google Network will consist of general posts and targeted ads using Facebook Ads Manager. Partner newsletters and websites include Hamilton Academy of Medicine, McMaster Institute for Research on Aging, McMaster Okanagan Charter, and RSearch. We will also engage clinicians and their administrative staff within the McMaster Department of Medicine, Hamilton Health Sciences outpatient clinics, Queen Square Family Health, and other community, primary, and specialty clinics in Ontario to identify potential participants and invite them to register with Trial My App to determine if they want to participate in the trial. Snowball sampling through participants and personal networks may also be used. Interested candidates will be provided a link or a quick response code to access the website within the marketing materials. They could also contact our research assistant through a dedicated Trial My App email account if they prefer the initial contact by email or phone.

**Figure 1:**
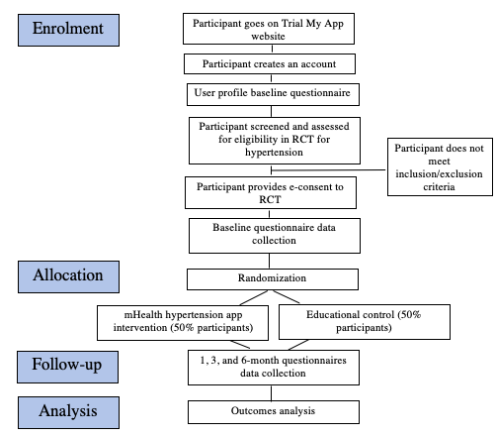
Participant flow diagram. RCT: Randomized controlled trial; mHealth: mobile health.

**Figure 2:**
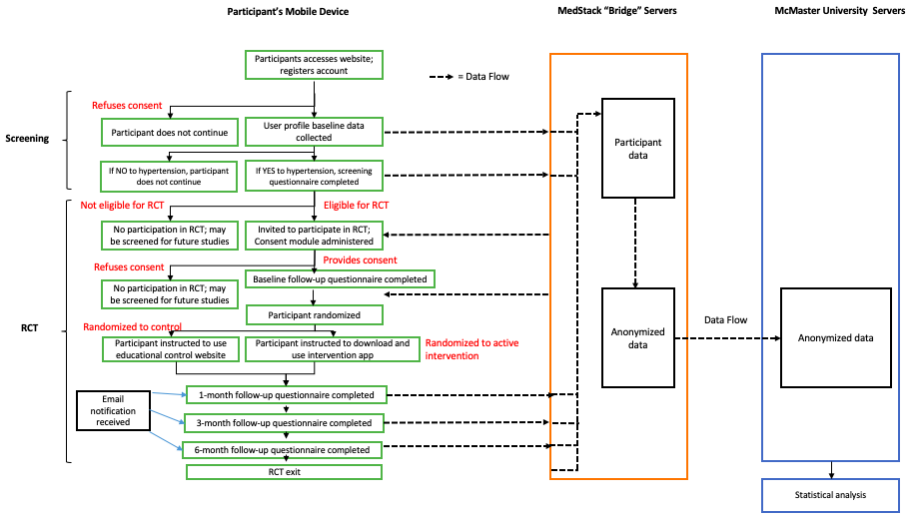
Trial My App web flow diagram. RCT: randomized controlled trial.

All trial stages, including screening, consent, randomization, and collection of clinical and patient-important outcomes data, will be performed virtually using the Trial My App platform. This phase includes a substudy to validate the web-based collection of patient data. Ethics approval of the substudy will be sought separately. Participants will be asked to register on the Trial My App site with an email and a password. The informed consent form is in Multimedia Appendix 2. Once they have electronically consented to using the web app, they will complete a user profile questionnaire and be screened for participation in the pilot trial in a subsequent survey. Multimedia Appendix 3 contains all user baseline, screening, and follow-up questionnaires. If participants meet inclusion criteria, they will be asked to electronically consent to take part in the pilot trial and provide data at 0, 1, 3, and 6 months. The participant flow is shown in Figure 1. The diagram of the app flow is shown in Figure 2.

### Sample Size

In the feasibility study, at least 80% of the participants in each group who successfully complete the final questionnaire within 1 year of trial start will be considered as the primary feasibility outcome. To estimate a completion rate of 80% in each group within a margin of error of 8% and with a confidence interval of 95%, we estimated a sample size of at least 100 participants in each group, and 200 participants in total is required. With a sample size of 200 participants in total (ie, 100 per group), we will have 80% power to detect a reduction in blood pressure of 8 mm Hg, which is considered a minimally clinically important difference. Assuming an 18 mm Hg standard deviation in systolic blood pressure, to detect an 8 mm Hg difference between groups with a power of 80% and a type I error of 5%, we would ideally require 81 participants in each arm [28]. Accounting for a 20% dropout rate, we aim to enroll 100 participants in each arm. We recognize that the selected app may not result in such a large difference in the systolic blood pressure, but the calculation is to guide our recruitment targets. If a difference in the blood pressure is found, we will be able to inform patients and physicians that this app may have a major impact on clinical outcomes such as heart attack, stroke, and congestive heart failure. 

### Intervention

After screening and baseline questionnaires, participants will be randomized using a web-based blocked randomization list of 4, 6, or 8 block sizes and a 1:1 allocation ratio. The intervention group will be instructed to download the Sphygmo BP app via a link provided within the Trial My App. The control group will receive a link to the Heart and Stroke foundation website, which includes information on hypertension management and measuring blood pressure [29]. All participants are expected to continue to receive usual care by their physician, including any anti-hypertensive medication and lifestyle changes.

### Data Collection

At 1, 3, and 6 months after enrollment into the RCT, Trial My App will send email reminders with a link to follow-up questionnaires to all participants to assess self-reported blood pressure and several patient-reported outcomes. Completing follow-up will be defined as answering the questionnaire within 7 days of receipt of the questionnaire notification via email. To encourage participation and reduce attrition, participants will receive a Can $10 (US $1=Can $1.27) electronic gift card for each completed assessment and an additional Can $10 gift card if they complete all 4 follow-up assessments.

### Outcomes

#### Feasibility Outcomes

Our primary goal at this stage is to determine whether the Trial My App platform can be used to conduct RCTs evaluating mHealth apps by the ability to complete an adequately powered RCT of least 80 participants in each group that successfully complete the study and the 6-month questionnaire within an arbitrarily defined reasonable time frame of 12 months. The remaining feasibility outcomes and associated indicators are shown in Table 1.

**Table 1:** Feasibility outcomes and indicators.

Outcome	Definition	Indicator	Minimum required sample size
Participation completion	Number of participants who successfully complete the final questionnaire within 1 year of trial start	80% out of total randomized participants in each group	100 participants per group is needed to achieve a margin of error of 8% with a 95% confidence level
Eligibility	Proportion of participants who sign up that meet eligibility criteria	At least 50% of responses to baseline and screening questionnaires are eligible	200 participants in total to achieve a margin of error of 7% with a 95% CI
Recruitment	Number of eligible participants recruited and consented	At least 50% of target sample size of 200 randomized within 6 months	200 participants in total to achieve a margin of error of 7% with a 95% CI
Retention	Proportion of withdrawal and dropouts after recruitment	Less than 20% of the participants lost to 6-month follow-up	200 participants in total to achieve a margin of error of 5.5% with a 95% CI
Outcome acceptability	Follow-up questionnaire completion rates	70% of the questionnaires that are submitted within 7 days of notification reminder	200 participants in total to achieve a margin of error of 6.3% with a 95% CI
Intervention acceptability	Frequency of app usage in the intervention group	Answers to frequency of use and features used questions in follow-up questionnaires	N/A^a^
Appropriateness of data collection processes	Completeness of data	50% of the questionnaires completed and less than 20% of the missing response rates in each questionnaire	N/A

^a^N/A: not applicable.

#### Pilot Efficacy Outcomes

The secondary objective of the RCT is to conduct a trial comparing the intervention hypertension app with a control group. The main outcome is clinical changes in blood pressure based on self-reported answers in the questionnaires. The remaining patient-reported outcomes and their associated indicators from baseline to 6 months are shown in Table 2. Adherence to hypertension self-care behaviors will be scored using the validated H-SCALE (Hypertension Self-Care Activity Level Effects) [30] and health care self-efficacy will be scored using the validated Health Confidence Score [31]. Similar outcome measures (eg, medication adherence, diet, physical activity) have been used in other studies of hypertension apps and measure hypertension-specific self-efficacy scores [32].

**Table 2:** Efficacy outcomes and indicators.

Outcome, definitions		Indicator
**Clinical assessment**		
	Difference in mean change in blood pressure from baseline to 6 months between groups	Statistically significant difference in mean change in systolic blood pressure measurements (defined as *P*<.05 using a Pearson test)
	Proportion of patients at their recommended blood pressure	Blood pressure measurements compared to standard ranges
**Self-management ability**		
	Difference in mean change of self-managing behaviors	H-SCALE^a^ score and statistically significant correlations with blood pressure at 95% CI
	Difference in mean change in feelings of self-efficacy	Frequency distribution and mean of Health Confidence Score at 95% CI
**Patient-reported outcomes**		
	Descriptive analysis of patient-oriented experiences between groups at baseline and 6 months	Agreeability with goal setting, decision making, sharing data, and empowerment statements in questionnaires developed from the advisory board themes (at 95% CI)

^a^H-SCALE: Hypertension Self-Care Activity Level Effects.

### Statistical Analysis

Descriptive statistical analysis will be performed on the data set using appropriate statistical methods to measure feasibility. Efficacy outcomes will be compared between the intervention and control groups by using logistic regression.

### Ethics Approval

This study has received approval from the Hamilton Integrated Research Ethics Board, #8039. This is a minimal risk study and the subject matter is not likely to be distressing to participants. Participation in this study may be inconvenient, taking about 10-20 minutes to complete at each timepoint (baseline, 1, 3, and 6 months). In the event of possible emotional distress, participants will be able to discontinue the use of the web app. Participant identifiers will be replaced with a code number; therefore, the data that researchers will access are not identifiable. As the intervention app is designed to track blood pressure to facilitate management and communication and participants will continue to receive usual care, no other harms are foreseen. Participants will be asked to complete an electronic consent form. The research team will have access to the final trial data set and ensure that all privacy policies are strictly maintained. Data will be securely stored on a MedStack server built into the Trial My App platform. MedStack is a health data privacy compliance automation platform that builds, measures, and actively manages compliance and provides secure, flexible, and single-tenant cloud infrastructure tailored to Trial My App. Medstack complies with Ontario’s Personal Health Information Privacy Act legislation. The information collected will be anonymized and encrypted before transferring to a secure server and firewall-protected network on a password-protected computer located at the Health Information Research Unit at McMaster University.

## Results

### Trial Progress

The development of the Trial My App web platform has been completed with a software developer and has undergone functionality and remote usability testing to uncover technical bugs and improve the design. The creation of survey instruments has been completed in collaboration with our patient partners and advisory board. Recruitment is expected to begin in the first quarter of 2021; data collection and analysis are expected to be completed approximately 1 year after study commencement. Dissemination of results will occur through conferences and publications.

### Patient Engagement Strategy

Two patients with lived experience of cardiovascular diseases are collaborating as partners on the research team and additional patients serve on an advisory board overseeing the development of Trial My App. They have identified criteria for selecting apps to evaluate in future RCTs and aided in developing outcomes that are relevant to patients managing their cardiovascular risk factors with apps. Key contributions of our patient partners include joining bimonthly meetings with the research team to discuss project planning, developing questions for and taking part in the advisory group, testing the usability of Trial My App, reviewing and contributing to publications and other knowledge translation outputs, and contributing to production and circulation of recruitment materials. Any required training on these skills is provided by the research team.

## Discussion

To our knowledge, this study is the first of its kind to create a web-based platform to conduct RCTs of mHealth apps for cardiovascular risk. A limitation of this methodology is the collection of self-report data as it is subject to several response biases, including social desirability, recall, or measurement error biases. The research team will include an additional substudy to measure the concordance of self-reported blood pressure measurements submitted via the Trial My App web app and the reference standard of 7-day average home blood pressure measurements [33]. A subgroup of participants will measure their blood pressure by using identical automatic home blood pressure monitoring devices 4 times daily for 1 week. The research team anticipates that participants will use the apps to varying degrees to help them manage their health, as they would normally; assessing these elements are beyond the scope of the study. The investigators expect that the pilot findings will demonstrate the feasibility of gathering valid patient-reported outcomes via web-based questionnaires that can be applied more broadly to other clinical studies. The findings of this trial may inform the evaluation of other mHealth apps for other conditions at a relatively low cost and more quickly than using traditional RCT methods. These results will also provide useful information for app developers who are interested in testing their apps for clinical effectiveness as well as patients and clinicians who are interested in incorporating effective mHealth apps into their care.

## Appendix

Multimedia Appendix 1 Description of the blood pressure management app used for the study.

Multimedia Appendix 2 Information and consent form.

Multimedia Appendix 3 Study instruments.

Multimedia Appendix 4 Peer review report.

## Abbreviations

mHealth: mobile health
RCT: randomized controlled trial
