# Supplementary material for: Feasibility of a Web-Based Platform (Trial My App) to Efficiently Conduct Randomized Controlled Trials of mHealth Apps For Patients With Cardiovascular Risk Factors: Protocol For Evaluating an mHealth App for Hypertension
Source: JMIR Res Protoc. 2021 Feb 1;10(2):e26155. doi: 10.2196/26155 (PMC7884212; doi:10.2196/26155)

Sphygmo BP & Glucose is a free blood pressure and blood glucose tracking mobile application available on Android and iOS. It was developed by mmHg Inc., a company of doctors and engineers at the University of Alberta. The app is recommended by Hypertension Canada.

There are several features found on the application. There are tabs for tracking blood pressure, glucose, weight, temperature, respiratory rate, and oxygen saturation. There is a tab for hypertension education on how to manage high blood pressure and what kinds of devices a user should choose. Blood pressure readings can be inputted with Bluetooth blood pressure monitors or manually entered. Readings can also be entered by scanning the QR code or barcode on the result printout of a blood pressure kiosk. The app will provide 1, 7, and 30-day trends and averages of blood pressure readings. A unique feature of this app is the ability to link an account with a healthcare provider for telemonitoring support and the remote assessment of blood pressure. Users must sign a privacy and user agreement upon registration. Users may backup and export data.

Images of the user interface below are provided by the developers in the app store. Sphygmo BP has an easy to navigate and attractive user interface.


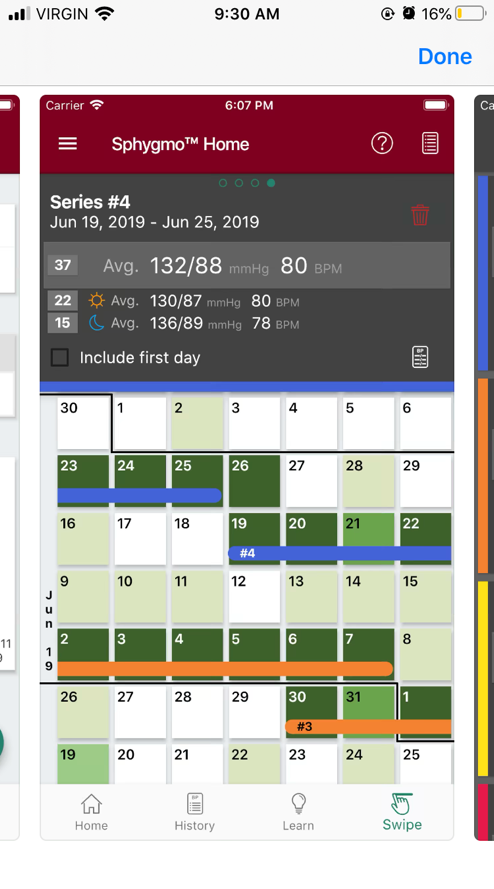

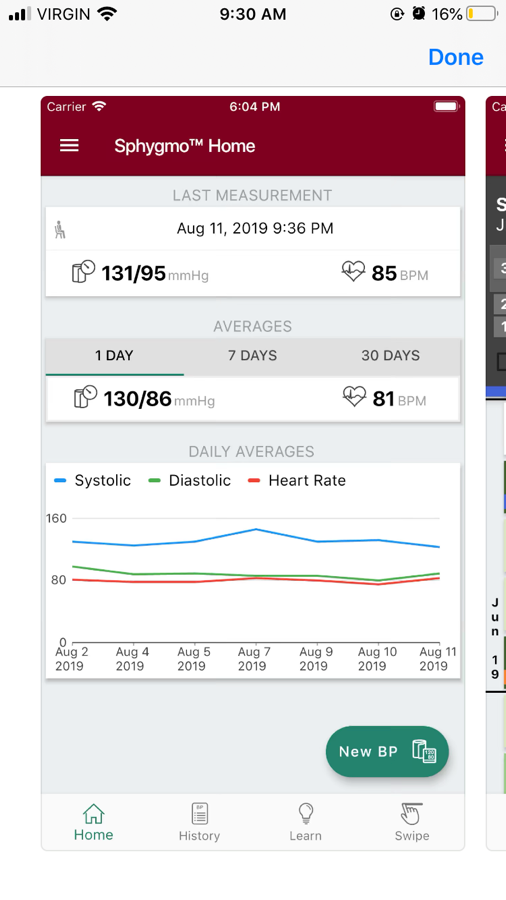

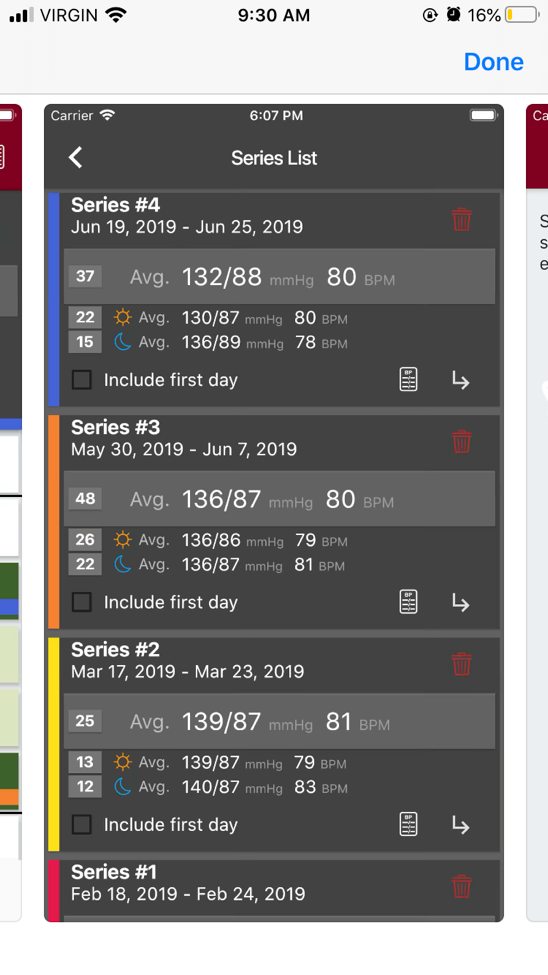

Supplement: Multimedia Appendix 1 [file resprot_v10i2e26155_app1.docx]
