# Supplementary material for: Feasibility of a Web-Based Platform (Trial My App) to Efficiently Conduct Randomized Controlled Trials of mHealth Apps For Patients With Cardiovascular Risk Factors: Protocol For Evaluating an mHealth App for Hypertension
Source: JMIR Res Protoc. 2021 Feb 1;10(2):e26155. doi: 10.2196/26155 (PMC7884212; doi:10.2196/26155)
Supplement: Multimedia Appendix 2 [file resprot_v10i2e26155_app2.docx]

**PARTICIPANT LETTER OF INFORMATION/CONSENT TO RCT**

Feasibility of a web-based platform to efficiently conduct RCTs of mHealth apps for CV risk factors: Protocol for the Trial My App hypertension RCT

**Principal Investigator**: Dr. Cynthia Lokker, McMaster University, Faculty of Health Sciences, [apptrial@mcmaster.ca](mailto:lokkerc@mcmaster.ca), 905-525-9140 x22208

Funding source: Canadian Institutes of Health Research

Thank you for answering our screening questions. You are eligible for a trial on a blood pressure management app. The following sections will explain what is involved with this study, so you can decide whether it is right for you.

**Invitation to participate in a research study**

We are inviting you to participate in a randomized controlled trial on a blood pressure management app. In order to decide whether you want to be a part of this research study, you should understand what is involved and the potential risks and benefits. The following sections will provide detailed information about the research study. Once you understand the study, you will be asked to sign this form if you wish to participate. Please take your time to make your decision. Feel free to discuss it with your friends and family, or your family physician.

**Why is this research being done?**

Patients with high blood pressure need to regularly monitor their readings. Some choose to use blood pressure management smartphone apps to help while others rely on other methods. However, we have very little evidence about whether these applications really do help. The goal of our study is to test the feasibility of using our new platform to conduct randomized controlled trials of these apps.

This study will randomly assign you to one of two blood pressure management strategies, a tracking app or informational website. Over a 6-month period, we will ask you about your experience and whether you were successful at managing your blood pressure. In this way, our second objective is to evaluate if one strategy is better than another.

**What will my responsibilities be if I participate in this study?**

You may be asked to use a blood pressure management app for 6 months to track your blood pressure or to another blood pressure resource. You will be asked survey questions after 1 month, 3 months, and 6 months; each time this will take about 10-20 minutes. You may also be asked to participate in another component of the study to gather blood pressure readings.

**Potential benefits**

The blood pressure management strategy which you are asked to use may help you control your blood pressure. Your participation will also help researchers understand if one strategy is better than another. This will allow more people to use the best approaches for blood pressure management and may allow software developers to make apps more effective. We hope that data from this study will help improve health for all people trying to manage their blood pressure.

**Issues to consider**

Some questions may make you uncomfortable. If this happens, simply do not respond to the question. You may be asked questions about your health, fitness, and activity. This may have an effect on your emotions.

**Will there be any costs?**

Participation in this study will involve data transmission using your smartphone. This may incur costs from your cellular service provider.

**Will I be paid to participate in this study?**

You will receive an electronic $10 gift card after the completion of each survey (at the start of the trial, 1 month, 3 months, 6 months). This is to reimburse you for your time and cost of data transmission through your smartphone. If you complete all 4 surveys, you will receive an additional $10 gift card.

**Data usage**

Any data that we collect will be encrypted and sent to a secure database, with your name replaced by a random code. Collected data may allow researchers, as well as you, to understand patterns and details about health. Your coded study data will be used for research by McMaster University and may be shared to other researchers approved by McMaster University.

**Risk to Privacy**

We will make every effort to protect your information, but total anonymity cannot be guaranteed. Your data will not be shared with anyone except with your consent or as required by law. To help ensure confidentiality, all data we gather will be encrypted using similar technology as is used in Internet banking. All personal information such as your name will be removed from the data once collection is complete and will be replaced with a study code number prior to analysis. A list linking the number with your name will be kept in a separate, secure database. The data, with identifying information removed will be securely stored in MedStack’s databases which is PHIPA compliant.

For the purposes of ensuring the proper monitoring of the research study, it is possible that a member of the Hamilton Integrated Research Ethics Board and representatives of McMaster University may consult your research data. However, no records which identify you by name or initials will be allowed to leave the secure databases. By signing this consent form, you or your legally acceptable representative authorize such access.

**Withdrawing**

Your participation in the study is voluntary. You may withdraw your consent and discontinue participation from the trial at any time. We will not collect or store any new data if you choose to withdraw but you cannot remove the data you have already submitted. To withdraw from the study, simply select "Leave Study" on the profile tab.

**Information About the Study Results**

The results from this study may be used in journal articles, presentations, or books. Confidentiality will be respected in each of these contexts; we will not use your name or any information that would allow you to be identified.

Conflicts of interest

The investigators and McMaster University have created and own the intellectual property of Trial My App.

**Questions?**

If you have any questions about the research now or later, or if you think you have a research-related injury, please contact Cynthia Lokker at apptrial@mcmaster.ca, 905-525-9140 x22208. This study has been reviewed by the Hamilton Integrated Research Ethics Board #8039. If you have any questions about your rights as a research participant, please call the Office of the Chair, Hamilton Integrated Research Ethics Board at 905.521.2100 x 42013.

Please electronically sign below if you consent to participate in this trial.

____ I consent button

____ I do not consent button

_____________________________ Date
