# Supplementary material for: Feasibility of a Web-Based Platform (Trial My App) to Efficiently Conduct Randomized Controlled Trials of mHealth Apps For Patients With Cardiovascular Risk Factors: Protocol For Evaluating an mHealth App for Hypertension
Source: JMIR Res Protoc. 2021 Feb 1;10(2):e26155. doi: 10.2196/26155 (PMC7884212; doi:10.2196/26155)
Supplement: Multimedia Appendix 3 [file resprot_v10i2e26155_app3.pdf]

Stage 1: User Profile Baseline Questionnaire

1. What is your age?
   - ____ years
2. What is your sex?
   - Male
   - Female
   - Other
3. What is your weight?
   - In kg/lb
4. What is your height?
   - In cm/feet, inches
5. Has your doctor told you that you have high cholesterol?
   - Yes
   - No
   - Not sure
6. Has your doctor told you that you have High Blood Pressure?
   - Yes
   - No
7. Are you on treatment for high blood pressure?
   - Yes
   - No
8. Has your doctor told you that you have Diabetes?
   - Yes
   - No
9. Are you a current smoker?
   - Yes
   - No
10. Have you had a heart attack or coronary stent?
    - Yes
    - No
11. Have you had a stroke?
    - Yes
    - No
12. Has your doctor told you that you have narrowing of leg arteries (peripheral artery disease)?
    - Yes
    - No
13. Did your biological mother or father have a heart attack at an early age (younger than 65 for mother, younger than 55 for father)?
    - Yes
    - No
    - I don't know
14. Do you have a smartphone or tablet with internet connection for your own personal use?
    - Apple iPhone/iPad
    - Android
    - Blackberry
    - Windows
    - No I do not have a phone or tablet

Stage 2: Hypertension Screening Questionnaire Algorithm

[If they checked **YES to Hypertension** question, then they are potentially eligible for RCT]

"You may be eligible for a current trial for hypertension! To find out if you are eligible, we will need blood pressure measurements from the last 14 days, done in a way that can be easily repeated in future (for example, with your own blood pressure machine at home, or using a blood pressure machine at a drug store)."

1. Are you currently using an app for monitoring your BP?
   - Yes
     - Exclude. Prompt: "Thanks for this information. You are not currently eligible for this trial, but may be eligible for future studies. We will contact you as new studies are available." *Exit to Dashboard*
   - No
     - User continues to next question

2. Please confirm that you have a diagnosis of high blood pressure (hypertension)
   - No
     - Exclude. Prompt: "Thanks for this information. You are not currently eligible for this trial, but may be eligible for future studies. We will contact you as new studies are available." *Exit to Dashboard*
   - Yes
     - User continues to next question

[For patients who checked YES to Diabetes in Stage 1 Baseline Questionnaire]

3A. Please confirm that you have diabetes.
   - No, I do not have diabetes
     - User profile will automatically update
     - User continues to next question
   - Yes, I have diabetes
     - User continues to next question

[For patients who checked NO to Diabetes in Stage 1 Baseline Questionnaire]

3B. Please confirm that you have diabetes.
   - No, I do not have diabetes
     - User continues to next question
   - Yes, I have diabetes
     - User profile will automatically update
     - User continues to next question

[Next set of questions for all]

4. Do you live in Canada?
   - Yes

    i. User will continue to next question
- No
    i. Exclude. Prompt: "Thanks for this information. You are not currently eligible for this trial, but may be eligible for future studies. We will contact you as new studies are available." *Exit to Dashboard*
    ii.

5. Do you have access to a blood pressure measurement device at home or the drug store for your personal use (not in a clinic)?
    - Yes
        i. User will continue to next question
    - No
        i. Exclude. Prompt: "Thanks for this information. You are not currently eligible for this trial, but may be eligible for future studies. We will contact you as new studies are available." *Exit to Dashboard*

6. Do you have access to a smartphone/tablet with internet connection?
    - Yes
    - No, I don't have a smartphone or tablet/iPad
        i. Exclude. Prompt: "Thanks for this information. You are not currently eligible for this trial, but may be eligible for future studies. We will contact you as new studies are available." *Exit to Dashboard*].

7. Are you currently pregnant?
    - Yes.
        i. Exclude. Prompt: "Thanks for this information. You are not currently eligible for this trial, but may be eligible for future studies. We will contact you as new studies are available." *Exit to Dashboard*
    - No
        i. User continues to next question

8. Within the past two weeks, have you had a blood pressure measurement reading **<u>greater than or equal to</u>** 180 systolic (top number) or 120 diastolic (bottom number)?
    - Yes
        i. Exclude. Prompt: "You may have emergent hypertensive concerns and should seek medical attention. You are not currently eligible for this trial, but may be eligible for future studies. We will contact you as new studies are available." Exit to Dashboard.
    - No
        i. User moves onto next question

9. We will now ask you to measure your blood pressure using your usual measurement device (At home or at the pharmacy, not in the doctor's office) before proceeding.

Please follow these steps to ensure a correct reading:

- Don't drink caffeinated or alcoholic beverages, exercise, or smoke 30 minutes before measurement
- Rest for at least 5 minutes before starting
- Sit in a chair with your back straight and supported, with your feet flat on the ground
- Keep your arm supported on a flat surface so your elbow is at heart level
- Do not talk during the measurement
- Take 3 separate readings at least 1 minute apart and record all three results

You can come back to this question if you cannot enter your readings right now.

- First reading
  - i. Systolic (top number): ___
  - ii. Diastolic (bottom number): ___
- Second reading
  - i. Systolic (top number): ___
  - ii. Diastolic (bottom number): ___
- Third reading
  - i. Systolic (top number): ___
  - ii. Diastolic (bottom number): ___

Stage 3: Time 0 - Start of Trial Questionnaire

“The following questions help us understand your condition and perceptions before we start the trial. It should take about 20 minutes for you to complete the assessments”

How did you hear about Trial My App?

- o My primary care clinic
- o My hypertension clinic
- o The internet
- o Other

How did you take your blood pressure measurement in the previous questionnaire?

- o At my home
- o At another location (e.g. drug store)

How often do you check your blood pressure?

- o Daily
- o Most days
- o Once a week
- o Less than once a week

<u>H-SCALE, 33 questions</u>

<u>Health Confidence Score: 4-point scale</u>

<u>Trial My App Questions: 5-point scale</u>

The following questions are about how you manage your health and health behaviours.

How much do you agree with the following statements?

1. I feel I have the information I need to make decisions about my health
   - Strongly agree = 4
   - Agree = 3
   - Neutral = 2
   - Disagree = 1
   - Strongly disagree = 0

2. If I make a change to my health management, I know whether it was helpful or not
   - Strongly agree = 4
   - Agree = 3
   - Neutral = 2
   - Disagree = 1
   - Strongly disagree = 0

3. I sometimes change my health behaviours when the changes I have made are not working well for me
   - Strongly agree = 4
   - Agree = 3
   - Neutral = 2
   - Disagree = 1
   - Strongly disagree = 0

4. I am able to set personal goals for my health
   - Strongly agree = 4
   - Agree = 3
   - Neutral = 2
   - Disagree = 1
   - Strongly disagree = 0

5. I know when I am progressing towards my health goals
   - Strongly agree = 4
   - Agree = 3
   - Neutral = 2
   - Disagree = 1
   - Strongly disagree = 0

6. I feel good when I see progress towards my goals
   - Strongly agree = 4
   - Agree = 3
   - Neutral = 2
   - Disagree = 1
   - Strongly disagree = 0

7. I know when I am doing things that are good for my health
   - Strongly agree = 4
   - Agree = 3
   - Neutral = 2
   - Disagree = 1
   - Strongly disagree = 0

8. I have the information I need to understand my current state of health
   - Strongly agree = 4
   - Agree = 3
   - Neutral = 2
   - Disagree = 1
   - Strongly disagree = 0

9. I have the ability to make positive changes in my health

- Strongly agree = 4
- Agree = 3
- Neutral = 2
- Disagree = 1
- Strongly disagree = 0

10. I share my health goals and progress with my health care provider (doctor, nurse, pharmacist, etc.)
    - Strongly agree = 4
    - Agree = 3
    - Neutral = 2
    - Disagree = 1
    - Strongly disagree = 0
11. I am satisfied with the level of communication with my health care provider (doctor, nurse, pharmacist, etc.)
    - Strongly agree = 4
    - Agree = 3
    - Neutral = 2
    - Disagree = 1
    - Strongly disagree = 0
12. I would be interested in participating in another part of the study measuring blood pressure and would like to be contacted for more information.
    - Yes → enter email address: ______
    - No

Stage 4: 1- and 3-month Questionnaire

"Hello from the team at Trial My App! It is time for you to complete a series of questionnaires about your blood pressure. Before you start, please be ready to measure your blood pressure, ideally using the same measurement device and location that you normally use."

<u>Blood Pressure</u>

1. We will now ask you to measure your blood pressure using your usual measurement device (At home or at the pharmacy, not in a doctor's office) before proceeding.

Please follow these steps to ensure a correct reading:

- Don't drink caffeinated or alcoholic beverages, exercise, or smoke 30 minutes before measurement
- Rest for at least 5 minutes before starting
- Sit in a chair with your back straight and supported, with your feet flat on the ground
- Keep your arm supported on a flat surface so your elbow is at heart level
- Do not talk during the measurement
- Take 3 separate readings at least 1 minute apart and record all three results

You can come back to this question if you cannot enter your readings right now.

a. First reading
   i. Systolic (top number): ___
   ii. Diastolic (bottom number): ___
b. Second reading
   i. Systolic (top number): ___

ii. Diastolic (bottom number): ___

c. Third reading

i. Systolic (top number): ___

ii. Diastolic (bottom number): ___

2. How did you take that measurement?
   - At my home
   - At another location (e.g. drug store)
3. How often do you check your blood pressure?
   a. Daily
   b. Most days
   c. Once a week
   d. Less than once a week

[For participants in the app arm of the trial]

<u>App Use Questions</u>

4. How frequently do you use the app?
   a. Daily
   b. Most days
   c. Weekly
   d. Less than weekly
5. What features of the app do you use? Check all that apply.
   a. Blood pressure measurement tracker
   b. Average blood pressure trends
   c. Telemonitoring
   d. Educational information about hypertension
   e. Other measurement tracking (for example, glucose, weight, temperature)

Stage 5: 6-month Questionnaire

"Hello from the team at Trial My App! It is time for you to complete the final series of questionnaires about your blood pressure. Before you start, please be ready to measure your blood pressure, ideally using the same measurement device and location that you normally use."

<u>Blood Pressure</u>

1. We will now ask you to measure your blood pressure using your usual measurement device (At home or at the pharmacy, not in a doctor's office) before proceeding.

Please follow these steps to ensure a correct reading:

- Don't drink caffeinated or alcoholic beverages, exercise, or smoke 30 minutes before measurement
- Rest for at least 5 minutes before starting
- Sit in a chair with your back straight and supported, with your feet flat on the ground
- Keep your arm supported on a flat surface so your elbow is at heart level
- Do not talk during the measurement
- Take 3 separate readings at least 1 minute apart and record all three results

You can come back to this question if you cannot enter your readings right now.

   d. First reading
      i. Systolic (top number): ___
      ii. Diastolic (bottom number): ___
   e. Second reading
      i. Systolic (top number): ___
      ii. Diastolic (bottom number): ___
   f. Third reading
      i. Systolic (top number): ___
      ii. Diastolic (bottom number): ___
2. How did you take your measurement?
   - At my home
   - At another location (e.g. drug store)
3. How often do you check your blood pressure?
   a. Daily
   b. Most days
   c. Once a week
   d. Less than once a week

<u>Trial My App Questions: 5-point scale</u>

The following questions are about how you manage your health and health behaviours.

How much do you agree with the following statements?

1. I feel I have the information I need to make decisions about my health
   - Strongly agree = 4
   - Agree = 3
   - Neutral = 2
   - Disagree = 1
   - Strongly disagree = 0

2. If I make a change to my health management, I know whether it was helpful or not
   - Strongly agree = 4
   - Agree = 3
   - Neutral = 2
   - Disagree = 1
   - Strongly disagree = 0

3. I sometimes change my health behaviours when the changes I have made are not working well for me
   - Strongly agree = 4
   - Agree = 3
   - Neutral = 2
   - Disagree = 1
   - Strongly disagree = 0

4. I am able to set personal goals for my health
   - Strongly agree = 4
   - Agree = 3
   - Neutral = 2
   - Disagree = 1
   - Strongly disagree = 0

5. I know when I am progressing towards my health goals

- Strongly agree = 4
- Agree = 3
- Neutral = 2
- Disagree = 1
- Strongly disagree = 0

6. I feel good when I see progress towards my goals
   - Strongly agree = 4
   - Agree = 3
   - Neutral = 2
   - Disagree = 1
   - Strongly disagree = 0

7. I know when I am doing things that are good for my health
   - Strongly agree = 4
   - Agree = 3
   - Neutral = 2
   - Disagree = 1
   - Strongly disagree = 0
8. I have the information I need to understand my current state of health
   - Strongly agree = 4
   - Agree = 3
   - Neutral = 2
   - Disagree = 1
   - Strongly disagree = 0
9. I have the ability to make positive changes in my health
   - Strongly agree = 4
   - Agree = 3
   - Neutral = 2
   - Disagree = 1
   - Strongly disagree = 0
10. I share my health goals and progress with my health care provider (doctor, nurse, pharmacist, etc.)
    - Strongly agree = 4
    - Agree = 3
    - Neutral = 2
    - Disagree = 1
    - Strongly disagree = 0
11. I am satisfied with the level of communication with my health care provider (doctor, nurse, pharmacist, etc.)
    - Strongly agree = 4
    - Agree = 3
    - Neutral = 2
    - Disagree = 1
    - Strongly disagree = 0

<u>H-SCALE – 33 questions, from time 0</u>

<u>Health Confidence Score: 4-point scale, from time 0</u>

[For participants in the app arm of the trial]

<u>App Use Questions</u>

1. How frequently do you use the app?

- Daily
- Most days
- Weekly
- Less than weekly

2. What features of the app do you use? Check all that apply.
   - Blood pressure measurement tracker
   - Average blood pressure trends
   - Telemonitoring
   - Educational information about hypertension
   - Other measurement tracking (for example, glucose, weight, temperature)

[For participants in the NON-app arm of the trial]

<u>App Use Questions</u>

1. In the last 6 months, have you started using a mobile phone app to track your blood pressure?
   - No
   - Yes → User continues to next questions
2. When did you start using the app?
   - Day/month/year calendar selection
3. How frequently do you use the app?
   - Daily
   - Most days
   - Weekly
   - Less than weekly
4. What features of the app do you use? Check all that apply.
   - Blood pressure measurement tracker
   - Average blood pressure trends
   - Telemonitoring
   - Educational information about hypertension
   - Other measurement tracking (For example, glucose, weight, temperature)
   - Other: ________

1. Do you feel that the app has helped you manage your health?
   - The app has helped me manage my health better
   - The app has not helped me manage my health any better than usual
2. What is the name of the app? _______
