# Supplementary material for: Feasibility of a Web-Based Platform (Trial My App) to Efficiently Conduct Randomized Controlled Trials of mHealth Apps For Patients With Cardiovascular Risk Factors: Protocol For Evaluating an mHealth App for Hypertension
Source: JMIR Res Protoc. 2021 Feb 1;10(2):e26155. doi: 10.2196/26155 (PMC7884212; doi:10.2196/26155)
Supplement: Multimedia Appendix 4 [file resprot_v10i2e26155_app4.pdf]

February 28, 2019

Canadian Institutes of Health Research / Instituts de recherche en santé du Canada

Notice of Decision / Avis de décision

Application Number/Numéro de la demande: 416969
Committee Code/Code du comité: SCT

Applicants/Candidats:	Lokker, Cynthia
With/Avec:	Bhagirath, Vinai C; Gabizon, Itzhak<br>Alvarez, Elizabeth; McGillion, Michael H; Schwalm, Jon-David R
Institution paid/<br>Établissement payé:	McMaster University
Title/Titre :	Refinement, usability testing, and feasibility of a mobile-based software infrastructure to conduct efficient RCTs of mobile health applications

Competition Outcome/Résultats du concours:

**Number in competition/<br>Nbre de demandes dans le concours:**	**28**
**Number approved/<br>Nbre de demandes approuvées:**	10

**Decision on your application/<br>Décision sur votre demande:**	**Approved / Approvée**
**Average annual amount/<br>Montant annuel moyen:**	**$99,161**
**Term/Durée:**	**1 Year(s), 0 Month(s)**

Peer Review Committee Recommendation, for your information and use/
Recommandation du comité d'examen par les pairs, pour fins d'information et d'utilisation :

Peer Review Committee Recommendation, for your information and use/
Recommandation du comité d'examen par les pairs, pour fins d'information et d'utilisation:

Committee/Comité: Catalyst Grant SPOR Innovative Clinical Trials

Number reviewed in that committee/Nbre de demandes examinées dans ce comité:	28
Number approved in that committee/Nbre de demandes approuvées dans ce comité:	10
Application rank within the committee/ Rang de la demande dans ce comité:	3
Percent Rank Within the Committee/ Rang en pourcentage au sein du comité:	92.5926
Rating/ Cote:	4.18

*** Applications receiving an overall rating of less than 3.5 will not be considered for funding. / Les demandes qui ont reçu une cote globale inférieure à 3.5 ne sont pas admissibles.

This document is for information only. Official payment is stated on the CIHR Authorization for Funding.
Document à titre d'information seulement. Le paiement officiel est indiqué sur l'autorisation de financement des IRSC.

Canadian Institutes of Health Research
160 Elgin Street, 9th Floor
Address Locator 4809A
Ottawa, Ontario K1A 0W9

Instituts de recherche en santé du Canada
160, rue Elgin, 9e étage
Indice de l'adresse 4809A
Ottawa (Ontario) K1A 0W9

Institute of Aging
Institute of Cancer Research
Institute of Circulatory and Respiratory Health
Institute of Gender and Health
Institute of Genetics
Institute of Health Services and Policy Research
Institute of Human Development and Child and Youth Health
Institute of Indigenous Peoples' Health
Institute of Infection and Immunity
Institute of Musculoskeletal Health and Arthritis
Institute of Neurosciences, Mental Health and Addiction
Institute of Nutrition, Metabolism and Diabetes
Institute of Population and Public Health

Institut du vieillissement
Institut du cancer
Institut de la santé circulatoire et respiratoire
Institut de la santé des femmes et des hommes
Institut de génétique
Institut des services et des politiques de la santé
Institut du développement et de la santé des enfants et des adolescents
Institut de la santé des Autochtones
Institut des maladies infectieuses et immunitaires
Institut de l'appareil locomoteur et de l'arthrite
Institut des neurosciences, de la santé mentale et des toxicomanies
Institut de la nutrition, du métabolisme et du diabète
Institut de la santé publique et des populations

February 28, 2019

Dr. Cynthia Lokker
McMaster University
Health Information Research Unit
Dept of Clinical Epidemiology & Biostatistics
1280 Main St W, CRL 137
Hamilton, Ontario L8S 4K1

Dear Dr. Lokker:

On behalf of the Canadian Institutes of Health Research (CIHR) we are pleased to inform you that your recent application submitted to the Catalyst Grant: SPOR Innovative Clinical Trials competition entitled, "Refinement, usability testing, and feasibility of a mobile-based software infrastructure to conduct efficient RCTs of mobile health applications" has been approved for funding. Documentation pertaining to the review of your application can be found through ResearchNet. Please note that your Authorization for Funding will follow in the mail.

As CIHR does not notify co-applicants of the decision, we ask that you inform those individuals involved, along with their research institutions (if different from your own), of the outcome of this application.

Should you require additional information, please contact the CIHR Contact Centre at support@cihr-irsc.gc.ca. Please do not contact the officers or members of the peer review committee.

Congratulations on your success in this competition.

Sincerely,

C. Leneï

Chaidwick Leneïs
Acting Manager, Program Design and Delivery
Research Programs Portfolio

481090-201811SCT-SCT-416969-150404-351A

CIHR IRSC
Discoveries for life / Découvertes pour la vie

Canada

Canadian Institutes of Health Research
160 Elgin Street, 9th Floor
Address Locator 4809A
Ottawa, Ontario K1A 0W9

Instituts de recherche en santé du Canada
160, rue Elgin, 9e étage
Indice de l'adresse 4809A
Ottawa (Ontario) K1A 0W9

Institute of Aging
Institute of Cancer Research
Institute of Circulatory and Respiratory Health
Institute of Gender and Health
Institute of Genetics
Institute of Health Services and Policy Research
Institute of Human Development and Child and Youth Health
Institute of Indigenous Peoples' Health
Institute of Infection and Immunity
Institute of Musculoskeletal Health and Arthritis
Institute of Neurosciences, Mental Health and Addiction
Institute of Nutrition, Metabolism and Diabetes
Institute of Population and Public Health

Institut du vieillissement
Institut du cancer
Institut de la santé circulatoire et respiratoire
Institut de la santé des femmes et des hommes
Institut de génétique
Institut des services et des politiques de la santé
Institut du développement et de la santé des enfants et des adolescents
Institut de la santé des Autochtones
Institut des maladies infectieuses et immunitaires
Institut de l'appareil locomoteur et de l'arthrite
Institut des neurosciences, de la santé mentale et des toxicomanies
Institut de la nutrition, du métabolisme et du diabète
Institut de la santé publique et des populations

February 28, 2019

Dr. Cynthia Lokker
McMaster University
Health Information Research Unit
Dept of Clinical Epidemiology & Biostatistics
1280 Main St W, CRL 137
Hamilton, Ontario L8S 4K1

Dear Dr. Lokker:

On behalf of the Canadian Institutes of Health Research (CIHR), I wish to congratulate you on your success in the recent CIHR funding competition!

As health researchers, we are united by a common goal: to improve the health and well-being of Canadians and people throughout the world. Through CIHR, the Government of Canada provides vital support to Canadian researchers, spanning the tightly linked pillars of health research, with the ultimate goal of improving health for all Canadians. As a recipient of this funding, you are now part of this endeavour.

As you are aware, the evaluation of your grant application was made possible thanks to peer reviewers who generously volunteer their time to support the Canadian health research enterprise. As a CIHR-funded researcher, your knowledge and expertise are invaluable to this process. If you are not already a peer reviewer, I would like to take this opportunity to invite you to become a member of the College of Reviewers. Please visit www.cihr-irsc.gc.ca/e/49923.html to learn more and to register for membership.

As health researchers, we are living at a time when science is under careful scrutiny, and it has become more important than ever to ensure that our work is clearly understood. As such, I would ask that you recognize your CIHR funding when delivering presentations and communicating about your research, and that you continue to advocate for the critical importance of research in advancing the health of Canadians.

Once again, please accept my congratulations on this impressive achievement and my best wishes for success in all your endeavours. I look forward to following the progress of your research.

Sincerely,

Michael J. Strong, MD, FRCPC, FAAN, FCAHS
President

481091-201811SCT-SCT-416969-150404-CONGR

CIHR IRSC
Discoveries for life / Découvertes pour la vie

Canada

Review Type/Type d'évaluation: Committee Member 1/Membre de comité 1
Name of Applicant/Nom du chercheur: Lokker, Cynthia
Application No./Numéro de demande: 416969
Agency/Agence: CIHR/IRSC
Competition/Concours: 2018-11-14 Catalyst Grant: SPOR Innovative Clinical Trials/Subvention catalyseur: Essais cliniques novateurs de la SRAP
Committee/Comité: Catalyst Grant SPOR Innovative Clinical Trials/Subvention catalyseur: Essais cliniques novateurs de la SRAP
Title/Titre: Refinement, usability testing, and feasibility of a mobile-based software infrastructure to conduct efficient RCTs of mobile health applications

---

Assessment/Évaluation:

1. Research Approach

· Extent to which the application responds to the objectives and relevant research areas of the funding opportunity.

Comments : The applicants propose to : (i) further develop and refine a mobile app and associated infrastructure (Trial My App) for trialing mobile health applications; (ii) co-develop with patient partners and advisors a guide to selecting apps to evaluate and identifying appropriate patient-oriented outcomes to measure in their evaluation; and (iii) evaluate the feasibility of using Trial My App to conduct a trial by carrying out a pilot trial of a hypertension-related app (goals are to validate self-reported app-collected outcome data and assess feasibility with respect to recruitment and retention of trial participants).

The proposed project is an excellent fit with the objectives of the funding opportunity, as it involves a pilot evaluation of a novel trial methodology. It is also relevant to the research areas as described in the funding opportunity.

· Originality of the proposed research, in terms of the hypotheses/research questions addressed, novel technology/methodology, and/or novel applications of current technology/methodology.

Comments : This is a highly innovative project that seeks to respond to key challenges in evaluating mobile health apps, including the speed of technological developments (leading to traditional trial results being obsolete/irrelevant by the time they are published) and the need for more cost-efficient evaluation strategies. The potential for a mobile app-based solution to address these challenges is enticing but largely untested to date. This catalyst project fills that gap.

**Review Type/Type d'évaluation:**	Committee Member 1/Membre de comité 1
**Name of Applicant/Nom du chercheur:**	Lokker, Cynthia
**Application No./Numéro de demande:**	416969
**Agency/Agence:**	CIHR/IRSC
**Competition/Concours:**	2018-11-14 Catalyst Grant: SPOR Innovative Clinical Trials/Subvention catalyseur: Essais cliniques novateurs de la SRAP
**Committee/Comité:**	Catalyst Grant SPOR Innovative Clinical Trials/Subvention catalyseur: Essais cliniques novateurs de la SRAP
**Title/Titre:**	Refinement, usability testing, and feasibility of a mobile-based software infrastructure to conduct efficient RCTs of mobile health applications

Assessment/Évaluation:

- Feasibility and appropriateness to apply the proposed design in answering the research question(s).

Comments : Trial My App has already been partially developed and is undergoing some usability testing. The technical aspects of securely collecting and storing data leverage IT expertise at the Population Health Research Institute (documented in the application and through support letters) and close to half the funding from the proposed catalyst grant will be for programming and server support during the app development and pilot study. This was reassuring as the technical aspects of the project are likely to be quite complex. The co-design of the guide with patient partners and advisors is an important strength of this project. It would have been helpful to see some additional details about how outcomes will be selected (in general and for the pilot trial). It is clear that the focus will be on patient-prioritized outcomes but I wonder whether standard criteria for the selection of outcomes and, in particular, outcome measurement instruments, will be applied (e.g., identifying potential tools that have established psychometric properties, could be generalizable across many mhealth applications, are feasible in length and format for completion on a smart phone, etc). The trial design is well-justified with respect to its goals around feasibility, recruitment and retention, and data quality.

- Appropriateness of the proposed strategies to address challenges and benefits associated with adopting non-traditional methods.

Comments : The applicants clearly identify the main challenges associated with this work, including validity of self-reported data, recruitment and retention, privacy issues, and engagement with the app being evaluated. With respect to self-reported data, the applicants will include a data quality cohort within the pilot trial. In this group, concordance between the self-reported data entered on the app and in-clinic assessment will be evaluated. This will be particularly important for outcomes that are not inherently based on the perspective of the participant, including, in the case of the pilot trial, blood pressure. It wasn’t clear to me how and when the data quality cohort participants would be invited to participate in this part of the work and I have some concern that these participants may interact differently with Trial My App if they are anticipating a data quality check. It may be possible to at least make some comparisons of the outcome data in this cohort vs the full cohort of trial participants.

**Review Type/Type d'évaluation:**	Committee Member 1/Membre de comité 1
**Name of Applicant/Nom du chercheur:**	Lokker, Cynthia
**Application No./Numéro de demande:**	416969
**Agency/Agence:**	CIHR/IRSC
**Competition/Concours:**	2018-11-14 Catalyst Grant: SPOR Innovative Clinical Trials/Subvention catalyseur: Essais cliniques novateurs de la SRAP
**Committee/Comité:**	Catalyst Grant SPOR Innovative Clinical Trials/Subvention catalyseur: Essais cliniques novateurs de la SRAP
**Title/Titre:**	Refinement, usability testing, and feasibility of a mobile-based software infrastructure to conduct efficient RCTs of mobile health applications

Assessment/Évaluation:

With respect to privacy, I wondered whether some additional legal/ethical analysis may be beneficial in considering whether and how this work could be scalable beyond Ontario/Canada, as is the goal of the applicants depending on the trial results (though understandably this may be beyond this funding application). One of the advantages of a mobile approach to evaluating mhealth apps is that there need not necessarily be a tie to a particular geographic location, clinic, or research centre. It will be important to understand the implications for future use of this technology if international studies are straightforward to implement with respect to privacy laws, etc.

· Strength, appropriateness and collective track record and expertise of the team to conduct the proposed project.

Comments : The lead PI is a junior investigator who has yet to establish a strong funding and publication track record. However, this work is based on a solid foundation of development of the idea and app and is supported by both experienced researchers and key organizations (particularly the Population Health Research Institute). The application was extremely clear, well-written, and well-supported by cited literature, which speaks to the quality and expertise of the applicant team.

· Appropriateness and feasibility of the approach and planned activities relative to the proposed budget.

Comments : The project is of appropriate scope; it is ambitious but feasible, with most of the funding proposed to support trainees and IT infrastructure.

**Review Type/Type d'évaluation:**	Committee Member 1/Membre de comité 1
**Name of Applicant/Nom du chercheur:**	Lokker, Cynthia
**Application No./Numéro de demande:**	416969
**Agency/Agence:**	CIHR/IRSC
**Competition/Concours:**	2018-11-14 Catalyst Grant: SPOR Innovative Clinical Trials/Subvention catalyseur: Essais cliniques novateurs de la SRAP
**Committee/Comité:**	Catalyst Grant SPOR Innovative Clinical Trials/Subvention catalyseur: Essais cliniques novateurs de la SRAP
**Title/Titre:**	Refinement, usability testing, and feasibility of a mobile-based software infrastructure to conduct efficient RCTs of mobile health applications

Assessment/Évaluation:

2. Potential Impact

· Potential impact on building patient-oriented research capacity.

Comments : I was pleased to see that trainee support and involvement was a priority for this application. Dr. Lokker has brought together a strong network of junior and senior investigators as well as patient partners. This project has excellent potential to build patient-oriented research capacity.

· Potential for the creation of new knowledge.

Comments : This is an innovative idea that addresses an important research gap.

· Quality of partnership building activities and potential to lead to sustainable relationships.

· Degree of meaningful inclusion of patients, health care professionals and policy makers and its appropriateness in relation to the goals of the proposal.

Comments : The patient engagement strategy for this project is impressive and incorporates current best practices. The two patient partners are well-connected to the topic and the team, based on their strong letters. They will receive training and will lead the meetings with the patient advisors, who in turn will be recruited in a manner that tries to achieve

**Review Type/Type d'évaluation:**	Committee Member 1/Membre de comité 1
**Name of Applicant/Nom du chercheur:**	Lokker, Cynthia
**Application No./Numéro de demande:**	416969
**Agency/Agence:**	CIHR/IRSC
**Competition/Concours:**	2018-11-14 Catalyst Grant: SPOR Innovative Clinical Trials/Subvention catalyseur: Essais cliniques novateurs de la SRAP
**Committee/Comité:**	Catalyst Grant SPOR Innovative Clinical Trials/Subvention catalyseur: Essais cliniques novateurs de la SRAP
**Title/Titre:**	Refinement, usability testing, and feasibility of a mobile-based software infrastructure to conduct efficient RCTs of mobile health applications

Assessment/Évaluation:

diversity in perspectives. The patient partners and advisors will be involved in a meaningful way from an early stage in the research and will receive some compensation in recognition of their work.

Review Type/Type d'évaluation: Committee Member 2/Membre de comité 2
Name of Applicant/Nom du chercheur: Lokker, Cynthia
Application No./Numéro de demande: 416969
Agency/Agence: CIHR/IRSC
Competition/Concours: 2018-11-14 Catalyst Grant: SPOR Innovative Clinical Trials/Subvention catalyseur: Essais cliniques novateurs de la SRAP
Committee/Comité: Catalyst Grant SPOR Innovative Clinical Trials/Subvention catalyseur: Essais cliniques novateurs de la SRAP
Title/Titre: Refinement, usability testing, and feasibility of a mobile-based software infrastructure to conduct efficient RCTs of mobile health applications

---

Assessment/Évaluation:

RESEARCH APPROACH:
This investigation team proposes to enable more efficient and rigorous evaluation of mHealth apps through the use of their new mobile research software “Trial my App”. Specifically, they are proposing to co-develop (with patients) a guide to select apps et chose outcomes, refine their software, and assess feasibility of its use in a RCT.

- The study premise is strong. The proposed methodology (“trial my App”) is innovative, highly relevant, and timely. Highly relevant with the current funding opportunity as this new technology as the potential to enable the conduct of rigorous, SPOR-oriented, comparative effectiveness or implementation trials in the future (major strengh).

- The proposed methods are aligned with the objectives and research questions, but felt short of expected details (i.e. conceptual or underpinning frameworks aside from iKT, precise study design [i.e.: conduct two meetings is not a study design], qualitative and quantitative analysis plan [i.e. non provided in either phases]) (major weakness).

- This is a highly qualified multidisciplinary team and environment to conduct the proposed trial. Knowledge users, including patients (principal KU), clinicians, and decision makers completes the team.

- Sex and gender is taken in consideration both from a methodological perspective and governance structure.

POTENTIAL IMPACT:

- There is an overall high potential to make a significant impact on patient experience of care or patient important outcomes as mHealth apps are abundant, extensively used by patients and recommended by clinicians, yet under evaluated.

- Strong, well described, sustainable partnerships with the appropriate knowledge users. Support of the Ontario SPOR Support Unit. The potential to built SPOR capacity, although not fully demonstrated by the investigation team, is there.

Review Type/Type d'évaluation: Committee Member 3/Membre de comité 3
Name of Applicant/Nom du chercheur: Lokker, Cynthia
Application No./Numéro de demande: 416969
Agency/Agence: CIHR/IRSC
Competition/Concours: 2018-11-14 Catalyst Grant: SPOR Innovative Clinical Trials/Subvention catalyseur: Essais cliniques novateurs de la SRAP
Committee/Comité: Catalyst Grant SPOR Innovative Clinical Trials/Subvention catalyseur: Essais cliniques novateurs de la SRAP
Title/Titre: Refinement, usability testing, and feasibility of a mobile-based software infrastructure to conduct efficient RCTs of mobile health applications

---

Assessment/Évaluation:

This proposal addresses an important challenge for assessing the efficacy of m-health apps: the cost /logistical difficulties of conducting RCTs. The investigators propose to develop a reusable platform for testing m-health apps and to test the use of this platform via an initial RCT for an app related to hypertension management.

The team is appropriately constituted to address both aspects of this initiative and will provide experiences for trainees to develop skills in this area of research. The study design is well constructed and includes elements, such as usability testing, that are not always a feature of similar proposals but should contribute significantly to the development of an effective platform for trials.

Potential issues include:

- There are major international efforts already in testing to accomplish similar functions. It is not clear from the proposal if the investigators are aware of these efforts and what unique value-add their efforts would bring in the context of competing platforms.

- Assuming that the trial demonstrates the viability of the platform, it is not clear if the team plans to make it available to other investigators beyond their current networks, although they do note plans to explore business models following the proof of concept. There do not appear to be plans or budget to support such an effort as part of this proposal, nor indications that the team plans to pursue this approach via other avenues. As early design decisions may affect future business models, proactive consideration of future uses may be helpful.

- Likewise, the team notes an intention to inform selection of apps for formularies and similar processes. Given previous research from Canada, the UK, and elsewhere that showed that efficacy is only one of a range of factors important to consumers in rating apps (others include factors such as security and cultural appropriateness), the team may wish to consider how their offering will complement other assessments. (Note: this suggestion is offered for the team's consideration only and did not affect the score of this proposal as it is beyond the scope of the current work.)

- The team may have overestimated the size of the population with hypertension, which could affect recruitment expectations for the initial trial. The proposal cites rates of 49% for men and 37% for women. 2014 Statistics Canada data suggest a rate of 17.7% for Canadians aged 12+. Rates do not exceed 40% until the 65+ age band.

Review Type/Type d'évaluation: Committee Member 4/Membre de comité 4
Name of Applicant/Nom du chercheur: Lokker, Cynthia
Application No./Numéro de demande: 416969
Agency/Agence: CIHR/IRSC
Competition/Concours: 2018-11-14 Catalyst Grant: SPOR Innovative Clinical Trials/Subvention catalyseur: Essais cliniques novateurs de la SRAP
Committee/Comité: Catalyst Grant SPOR Innovative Clinical Trials/Subvention catalyseur: Essais cliniques novateurs de la SRAP
Title/Titre: Refinement, usability testing, and feasibility of a mobile-based software infrastructure to conduct efficient RCTs of mobile health applications

---

Assessment/Évaluation:

1. Research Approach

· Extent to which the application responds to the objectives and relevant research areas of the funding opportunity

· Originality of the proposed research, in terms of the hypotheses/research questions addressed, novel technology/methodology, and/or novel applications of current technology/methodology.

Though I don't have much background on implementation science or comparative effectiveness research I would say this is a strong application which responds to the call for innovative research. I think there is real value in examining the feasibility and effectiveness of not an individual app but an evaluation tool for already existing apps. Healthcare apps are currently used in an unregulated and piecemeal way in patient care but have the potential to improve self-management, empower patients, provide real time and long term data and also be used collaboratively by patients and physicians. The researchers are therefore addressing a very pertinent gap by looking at how we can better implement apps directly into care in an evidence-based way.

· Feasibility and appropriateness to apply the proposed design in answering the research question(s).

Appropriateness of the proposed strategies to address challenges and benefits associated with adopting non-traditional methods.

The plan to first create criteria for app evaluation drawing on existing evidence and consultation with stakeholders and to then test both the feasibility as well as the effectiveness of using the app intervention in a pragmatic trial sounds like a prudent and conscientious approach. I cannot speak from experience about the quality of the strategy for testing the feasibility but as a patient I appreciate their not jumping straight to a large scale trial. Their inclusion of a component of the research which will validate the electronic data gathered by participants is appropriate . This is a key area of concern which must be addressed if we are to effectively integrate apps into patient care.

Review Type/Type d'évaluation: Committee Member 4/Membre de comité 4
Name of Applicant/Nom du chercheur: Lokker, Cynthia
Application No./Numéro de demande: 416969
Agency/Agence: CIHR/IRSC
Competition/Concours: 2018-11-14 Catalyst Grant: SPOR Innovative Clinical Trials/Subvention catalyseur: Essais cliniques novateurs de la SRAP
Committee/Comité: Catalyst Grant SPOR Innovative Clinical Trials/Subvention catalyseur: Essais cliniques novateurs de la SRAP
Title/Titre: Refinement, usability testing, and feasibility of a mobile-based software infrastructure to conduct efficient RCTs of mobile health applications

---

Assessment/Évaluation:

· Strength, appropriateness and collective track record and expertise of the team to conduct the proposed project.

N/A

· Appropriateness and feasibility of the approach and planned activities relative to the proposed budget.

See next section

2. Potential Impact

· Potential impact on building patient-oriented research capacity.

The proposal looks to involve patients at multiple levels and includes them not only in separate advisory groups but also as part of the core research team. The latter is key for building capacity for both researchers and patients to work together in partnership and to potentially give feedback on the engagement and partnership (“check-ins” were mentioned though not with both patients and other team members). A strengthening aspect is that there was descriptions of integrated KT with the patients including a mention of a “guide” to patient engagement based on what was learned through this project. If/how evaluation tools would be used in this would have been helpful to include. Also, given the number of “guides” o patient engagement in existence I think a critical analysis of their experience or how successful the strategies that the team implemented were would be more beneficial for knowledge building. The fact that I can comment at this level of detail about the knowledge translation/ dissemination/ capacity building plan reflects well on the proposal

· Potential for the creation of new knowledge.

**Review Type/Type d'évaluation:**	Committee Member 4/Membre de comité 4
**Name of Applicant/Nom du chercheur:**	Lokker, Cynthia
**Application No./Numéro de demande:**	416969
**Agency/Agence:**	CIHR/IRSC
**Competition/Concours:**	2018-11-14 Catalyst Grant: SPOR Innovative Clinical Trials/Subvention catalyseur: Essais cliniques novateurs de la SRAP
**Committee/Comité:**	Catalyst Grant SPOR Innovative Clinical Trials/Subvention catalyseur: Essais cliniques novateurs de la SRAP
**Title/Titre:**	Refinement, usability testing, and feasibility of a mobile-based software infrastructure to conduct efficient RCTs of mobile health applications

Assessment/Évaluation:

This criterium is pretty broad but referring to some of my first comments I think there is a need for better understanding of how to evaluate apps for use in care and also to examine and validate data gathered by these apps.

· Quality of partnership building activities and potential to lead to sustainable relationships.

See above but also positive to see that the lead patient advisors will receive training and support to actually facilitate the advisory group, test usability and will be involved at stages throughout the project. Excellent to read an actually explanation of how they will make decisions during the project (consensus model) since tensions between priorities and wants of different stakeholders is often a difficult part of engagement. I would have liked to have seen how other researchers who will be engaging would be oriented or supported to work with patient members as this is often novel for professionals and having a bit of prep for everyone can help facilitate relationship building and team work. Again, ongoing check-ins about the engagement not only with the patients (though great that they are doing this) but with the researchers can help address issues with partnership early and help contribute to understanding of how to work together (both for the individual projects benefit and for capacity building)

Comments :

· Degree of meaningful inclusion of patients, health care professionals and policy makers and its appropriateness in relation to the goals of the proposal.

Some of this is addressed above but I will add that there was obviously much consideration of engaging patients in multiple aspects of the project and also of engaging "lead" patient advisors who have relevant backgrounds and experiences to bring not just patient experience but also understanding of the technology and software development. It would have been helpful to read more about the development of the advisory group Given there are two patients built into the core team both of whom.

Given the described extent of involvement of the patients and their time commitment – the honorarium of

**Review Type/Type d'évaluation:**	Committee Member 4/Membre de comité 4
**Name of Applicant/Nom du chercheur:**	Lokker, Cynthia
**Application No./Numéro de demande:**	416969
**Agency/Agence:**	CIHR/IRSC
**Competition/Concours:**	2018-11-14 Catalyst Grant: SPOR Innovative Clinical Trials/Subvention catalyseur: Essais cliniques novateurs de la SRAP
**Committee/Comité:**	Catalyst Grant SPOR Innovative Clinical Trials/Subvention catalyseur: Essais cliniques novateurs de la SRAP
**Title/Titre:**	Refinement, usability testing, and feasibility of a mobile-based software infrastructure to conduct efficient RCTs of mobile health applications

Assessment/Évaluation:

$500/annum seems low. I would have though at least a living wage amount per hour (52 weeks) would lend more credence to the claims of value of the patient partnership. If the partners asked for this amount or declined higher that could have been noted. Also, there was no description of reimbursement for incurred costs by the two lead advisors or for travel/costs for any conferences or other KT events which were listed as part of the KT plan

Comments :

-

RATING

***NB*: Your rating should be within the range of your chosen descriptor (see Rating Scale below). We recommend that you decide on the range first, then the numeral.**

**Review Type/Type d'évaluation:**	Committee Member 5/Membre de comité 5
**Name of Applicant/Nom du chercheur:**	Lokker, Cynthia
**Application No./Numéro de demande:**	416969
**Agency/Agence:**	CIHR/IRSC
**Competition/Concours:**	2018-11-14 Catalyst Grant: SPOR Innovative Clinical Trials/Subvention catalyseur: Essais cliniques novateurs de la SRAP
**Committee/Comité:**	Catalyst Grant SPOR Innovative Clinical Trials/Subvention catalyseur: Essais cliniques novateurs de la SRAP
**Title/Titre:**	Refinement, usability testing, and feasibility of a mobile-based software infrastructure to conduct efficient RCTs of mobile health applications

Assessment/Évaluation:

Health applications have a significant potential to impact and enhance patient outcomes. However, we know little about how apps perform, or how patients experience them. This grant intends to develop a system to test mHealth apps, and to trial its framework on a particular app. There will be a process of patient consultation, and then there will be a feasibility phase to trial the Trial My App app evaluator app. Extremely thorough and well-thought-out grant application.

Research Approach

Extent to which the application responds to the objectives and relevant research areas of the funding opportunity.

Comments: Strong patient-oriented focus. Innovative on multiple levels: to use mHealth apps, to evaluate mHealth apps, to do so without traiditional RCTs. Creates a nice area for Canadian research competitiveness.

Originality of the proposed research, in terms of the hypotheses/research questions addressed, novel technology /methodology, and/or novel applications of current technology/methodology.

Comments: Original on multiple levels.

Feasibility and appropriateness to apply the proposed design in answering the research question(s).

Comments: Appears very feasible in one year.

Appropriateness of the proposed strategies to address challenges and benefits associated with adopting non-traditional methods.

Comments: Many potential imitations have been addressed in the grant, and accounted for.

Strength, appropriateness and collective track record and expertise of the team to conduct the proposed project.

Comments: The PI is a junior investigator, but an experienced individual with a relevant background. She has limited publications and experience leading academic projects, but the strength and thoroughness of this grant argues that she and her team will be able to lead this to fruition.

Review Type/Type d'évaluation: Committee Member 5/Membre de comité 5
Name of Applicant/Nom du chercheur: Lokker, Cynthia
Application No./Numéro de demande: 416969
Agency/Agence: CIHR/IRSC
Competition/Concours: 2018-11-14 Catalyst Grant: SPOR Innovative Clinical Trials/Subvention catalyseur: Essais cliniques novateurs de la SRAP
Committee/Comité: Catalyst Grant SPOR Innovative Clinical Trials/Subvention catalyseur: Essais cliniques novateurs de la SRAP
Title/Titre: Refinement, usability testing, and feasibility of a mobile-based software infrastructure to conduct efficient RCTs of mobile health applications

---

Assessment/Évaluation:

Appropriateness and feasibility of the approach and planned activities relative to the proposed budget.

Comments: Budget is reasonable, with major expenses being for grad students and for necessary technical elements.

Potential Impact

Potential impact on building patient-oriented research capacity.

Comments: Huge – patients use apps all the time, and this is an untapped resource for education, research and outcome modification.

Potential for the creation of new knowledge.

Comments: Excellent.

Quality of partnership building activities and potential to lead to sustainable relationships.

Comments: Excellent.

Degree of meaningful inclusion of patients, health care professionals and policy makers and its appropriateness in relation to the goals of the proposal.

Comments: Excellent.
